# UCK2-dependent conversion of cytidine to CTP is required for CTP uptake by *Chlamydia trachomatis*

**DOI:** 10.17912/micropub.biology.001607

**Published:** 2025-05-09

**Authors:** Laure Blanchet, Agathe Subtil

**Affiliations:** 1 UMR3691, Centre National de la Recherche Scientifique, Paris, Île-de-France, France; 2 Cell biology of microbial infection Unit, Institut Pasteur, Paris, Île-de-France, France; 3 Collège doctoral, Sorbonne Université, Paris, Île-de-France, France; 4 Université Paris Cité, Paris, Île-de-France, France

## Abstract

*Chlamydia trachomatis* , an obligate intracellular bacterium, develops into a vacuolar compartment called the inclusion. The bacteria import nucleoside triphosphates (NTPs) present in the inclusion lumen. It remains unclear whether nucleosides enter the inclusion lumen in their native form or as phosphorylated nucleotides. Using click chemistry coupled with fluorescence microscopy we provide evidence that cytidine requires phosphorylation by host uridine-cytidine kinase 2 (UCK2) prior to its incorporation into bacterial nucleic acids. These findings support the hypothesis that nucleosides are converted into nucleotides in the host cytoplasm prior to translocation into the inclusion lumen. Future work should therefore focus on the identification of nucleotide transporter(s) at the inclusion membrane.

**Figure 1. Silencing the expression of host UCK2 reduces the incorporation of 5-EC derivatives in C. trachomatis and does not affect bacterial proliferation:**
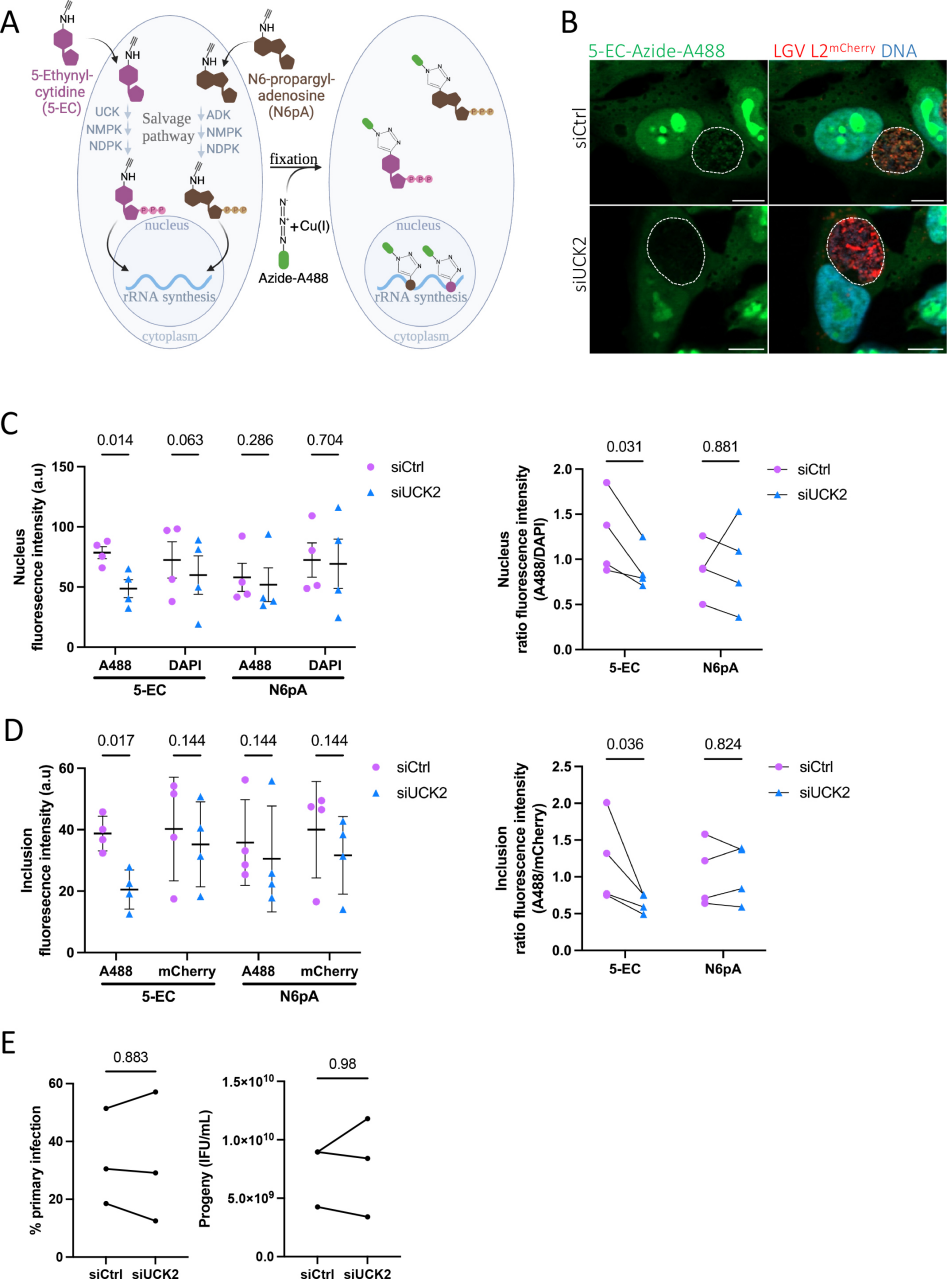
**(A)** Schematic view of the use of click chemistry to track nucleotide incorporation in nucleic acids. Cells are incubated with a nucleoside analog linked to an alkyne group (5-EC or N6pA). Nucleoside analogs are metabolized into nucleotides in the cytoplasm prior to incorporation into nucleic acids. After fixation and permeabilization, the click reaction between the alkyne and an azide group coupled to a fluorochrome, catalyzed by copper, allows to label the nucleotides. Created in https://BioRender.com. **(B)** HeLa cells treated with control (siCtrl) or siRNA targeting *UCK2* (siUCK2) were infected with *C. trachomatis* (LGVL2 ^mCherry^ ) for 16 h before adding 5-EC or N6pA. Cells were fixed 6 h later, permeabilized and processed for covalent staining of the probes with Alexa-488 (green) and of DNA with DAPI (blue). Bacteria appear in red. The dotted white lines delimit the inclusions. Bar = 10 μm. **(C-D)** *(Left)* Fluorescence intensities of nucleoside analogs and of DAPI (C) or mCherry (D), in the nuclei and inclusions, respectively, were quantified in 50 cells per experiments. a.u. arbitrary units. P-values were determined with a paired t-test. *(Right)* Fluorescence from the nucleoside analogs normalized to the DAPI or to the mCherry signals, respectively. P-values were determined with a ratio paired t-test. Means ± SD of four independent experiments are shown. **(E)** HeLa cells treated with siCtrl or siUCK2 were infected with LGV L2 ^GFP^ (multiplicity of infection (MOI) ~ 0.3) and the percent of infected cells was determined 24 h later. A duplicate well was used to determine IFUs produced 48 hpi. Three independent experiments are represented. P-values were determined with a paired t-test (primary infection) and a ratio paired t-test (progeny).

## Description

*Chlamydia trachomatis* is an obligate intracellular bacterium that infects human epithelial cells of the urogenital tract and of the ocular conjunctiva. It is the first cause of female infertility and of blindness of bacterial origin, representing a significant public health burden (Taylor, Burton, Haddad, West, & Wright, 2014; Van Gerwen, Muzny, & Marrazzo, 2022).

*C. trachomatis* develops within a membrane-bound compartment called the inclusion, located in the host cell cytoplasm. Upon co-evolution with its host *C. trachomatis* has lost genes for several biosynthetic pathways and has become dependent on the host for multiple metabolites (Stephens et al., 1998). Notably, the bacteria do not synthesize their own nucleoside triphosphates (NTPs), the building blocks of RNA and DNA (in the form of deoxynucleotides, dNTPs, for the latter), and acquire them from the host (Tipples & McClarty, 1993). Nucleosides and their phosphorylated derivatives, nucleotides, are transported across membranes only via specialized transporters. In mammalian cells, nucleosides, but not nucleotides, are imported from the extracellular environment through concentrative nucleoside transporters (CNTs) and equilibrative nucleoside transporters (ENTs) (Young, 2016). Once inside the cell, nucleosides are converted into NTP through the salvage pathway. *C. trachomatis* express two bacterial surface transporters, Npt1 and Npt2, that enable the uptake of NTPs, but not of nucleosides (Tjaden et al., 1999). The process by which host-derived NTPs become available for bacterial uptake within the inclusion lumen is unknown. This work aimed at testing the hypothesis that nucleosides needed to be converted to nucleotides before translocation into the inclusion lumen.

Click chemistry is commonly used to track the dynamics of nucleotide integration into DNA and RNA of eukaryotes (Fantoni, El-Sagheer, & Brown, 2021; Jao & Salic, 2008). Incorporation of nucleoside derivatives in nucleic acid polymers requires their conversion into nucleotides by dedicated enzymes. For instance, the cytidine derivative 5-ethynyl-cytidine (5-EC) needs to be converted into CMP by the uridine-cytidine kinase 2 (UCK2) before incorporation into RNA. It does not incorporate into DNA, likely because it is a poor substrate for the ribonucleotide reductase (Qu et al., 2013). We reasoned that if the conversion of 5-EC into CMP occurred prior to import in the inclusion, silencing *UCK2* should decrease the incorporation of 5-EC derived nucleotide in bacteria. Alternatively, if nucleosides were transported across the inclusion membrane, and conversion of 5-EC into CMP occurred in the inclusion lumen, silencing *UCK2* should not affect the 5-EC-derived signal associated with the bacteria. UCK2 expression was silenced using siRNA in HeLa cells before infection with a constitutively mCherry-expressing strain of *C. trachomatis* (LGV L2 ^mCherry^ ). At 16 hours post infection (hpi), 5-EC or N6pA (N6-propargyl-adenosine, a clickable analog of adenosine used as a negative control), were added to the medium culture for six hours, the samples were then fixed and processed for fluorescence microscopy, with the covalent fixation of a fluorophore to the probes (Figure 1.A). Fluorescence intensity of the probes was quantified in individual nuclei and inclusions. Fluorescence from DAPI and mCherry was used to estimate nuclear DNA content and bacterial load within inclusions, respectively. In the nucleus, the signal of nucleoside analogs mainly accumulated in structures corresponding to nucleoli, site of rRNA synthesis (Figure 1.B). Silencing of *UCK2* led to a 50% decrease of this nuclear signal for the cytosine derivative (Figure 1.B-C), while the nuclear signal from the adenosine derivative remained stable (Figure 1.C). This observation indicated that, as expected, silencing *UCK2* reduced the conversion of 5-EC into CTP in the host. The 5-EC derived signal was also observed in the inclusions as dots overlapping with bacteria (mCherry positive, Fig. 1B ). This finding suggests that the salvage pathway contributes to the nucleotide pool exploited by the bacteria, and that click chemistry is sensitive enough to detect NTP incorporation in *Chlamydia* . A 50% decrease in the 5-EC fluorescence signal in the inclusions was observed upon *UCK* 2 silencing (Figure 1.B and D), while the N6pA derived signal remained stable (Figure 1.D). This indicates that 5-EC is converted to CTP by UCK2 before being transported into the inclusion lumen. However, we cannot formally exclude that UCK2 itself is translocated inside the inclusion lumen, where the conversion could occur. Finally, we assessed whether *C. trachomatis* could complete its developmental cycle under these conditions. Reinfection assays revealed that *UCK2* silencing did not affect bacterial development (Figure 1.E). Thus, UCK2 depletion did not impair bacterial growth while significantly decreasing incorporation of 5-EC derived CTP in the bacteria. This implies that alternative source(s) of CTP compensate for the decrease in cytidine to CTP conversion upon *UCK2* silencing. *De novo* CTP biosynthesis in the host is likely solicited. The salvage pathway of UMP biosynthesis from uracil and phosphoribosyl pyrophosphate (PRPP) may also contribute, as *C. trachomatis* possesses a CTP synthase that can convert UTP into CTP (Wylie, Wang, Tipples, & McClarty, 1996).

In conclusion, our data indicate that the salvage pathway contributes to the nucleotide pools hijacked by the bacteria and that cytidine is converted to CTP before its translocation into the inclusion lumen. This implies the existence of NTP transporter(s) in the inclusion membrane, which could originate from the host or from the bacteria. Interestingly, several enzymes of purine biosynthesis were found in proximity to the inclusion membrane (Olson et al., 2019), indicating that *de novo* nucleotide biosynthesis might be manipulated by the bacteria to feed these elusive NTP transporters.

## Methods

**Cells and bacteria**

HeLa cells (ATCC) were grown in Dulbecco’s modified Eagle’s medium with Glutamax (Invitrogen), supplemented with 10 % (v/v) heat-inactivated fetal bovine serum and maintained at 37 °C, in 5 % CO _2_ atmosphere. *C. trachomatis* serovar LGV L2 strain 434 (obtained from ATCC) stably expressing mCherry (click chemistry) or the green fluorescent protein (progeny) were used (Agaisse & Derré, 2013). Bacteria were stored in sucrose-phosphate-glutamic acid buffer (SPG: 10 mM sodium phosphate [8 mM Na2HPO4- 2 mM NaH2PO4], 220 mM sucrose, 0.50 mM l-glutamic acid) at -80 °C (Scidmore, 2005; Vromman, Laverriere, Perrinet, Dufour, & Subtil, 2014).

**siRNA treatment**

100 000 HeLa cells were seeded in a 24-well plate. Eight hours later the cells were treated with a mix containing Lipofectamine RNAiMAX (Invitrogen) and 10 nM of control siRNAs (siCtrl, #SR-CL000-005) or siUCK2 (5′-GGGAUCUUGAGCAGAUUUUtt-3′) purchased from Eurogentec (Belgium), following the manufacturer’s recommendation. A second siRNA transfection was conducted 48 h after the first transfection for click chemistry assay or 2 h post infection for progeny assay. The silencing efficiency was confirmed by RT-qPCR 72 hours after the first siRNA treatment.

**Click chemistry**

30 000 cells treated twice, 72 h and 24 h earlier, with siRNA were seeded on coverslips in a 24-well plate. Eight hours later the cells were infected with LGV L2 ^mCherry^ (MOI = 1). Sixteen hpi 5 µM N6pA or 10 µM 5-EC were added to the culture medium and 6 hours later cells were washed and fixed in 4 % paraformaldehyde (w:v), 4 % (w:v) sucrose in PBS for 20 min. Cells were incubated for 10 min in 50 mM NH _4_ Cl in PBS and permeabilized in 0.3 % Triton X-100 in PBS for 10 min prior incubation for 1 h in a 25 µl drop containing 1 mM copper sulfate; 2.5 mM THPTA; 20 µM Azide-AF488; 12.5 mM acid ascorbic; and 0.5 µg/mL DAPI in PBS. Coverslips were mounted on slides with Mowiol (Sigma-Aldrich). Images were obtained on an Axio observer Z1 microscope equipped with an ApoTome module (Zeiss, Germany) 63× Apochromat lens. Images were taken with an ORCAflash4.OLT camera (Hamamatsu, Japan) using the Zen software from Zeiss. Images were analyzed with the Fiji software. The intensity fluorescence of the A488 fluorophore, DAPI, and mCherry were measured in the inclusion and the corresponding nuclei for approximately 50 cells per condition.

**Progeny assay**

100 000 HeLa cells were treated with siRNA in duplicate wells and infected 48 h later with LGV L2 ^GFP^ (MOI = 0.3) and a second siRNA transfection was performed 2 hpi. Twenty-four hpi cells were collected and the percent of primary infection was quantified by flow cytometry. Forty-eight hpi, bacteria from the duplicate well were collected by breaking the cells and used to reinfect in serial dilutions fresh HeLa cells plated in a 24-well plate. The next day, cells were collected from three wells infected at less than 30 % (estimated by visual inspection), the infection rate was determined by flow cytometry to deduce the number of IFUs present at 48 hpi. 20 000-30 000 events per samples were acquired on a CytoFLEX S (Beckman Coulter). Analysis was performed using FlowJo (version 10.0.7).

## Reagents

**Table:** 

**Product**	**Concentration stock**	**Origin**
**N6-propargyl-adenosine (N6pA)**	10 mM in DMSO	#CLK-N004-1, Jenabioscience
**5-ethynyl-cytosine (5-EC)**	100 mM in DMSO	#CLK-087, Jenabioscience
**azide-AF488**	50 mM in DMSO	#CLK-1275, Jenabioscience
**copper sulfate**	10 mM in H _2_ O	#209198, Sigma Aldrich
**acid ascorbic**	250 mM in H _2_ O	#A7631, Sigma Aldrich
**tris-hydroxypropyltriazolylmethylamine (THPTA)**	100 mM in DMSO	#762342, Sigma Aldrich
